# Tree-based approach for exploring marine spatial patterns with raster datasets

**DOI:** 10.1371/journal.pone.0177438

**Published:** 2017-05-16

**Authors:** Xiaohan Liao, Cunjin Xue, Fenzhen Su

**Affiliations:** 1 Institute of Geographical Science and Natural Resources Research, Chinese Academy of Sciences, Beijing, P.R. China; 2 Key Laboratory of Digital Earth Science, Institute of Remote Sensing and Digital Earth, Chinese Academy of Sciences, Beijing, P.R. China; 3 Key Laboratory of the Earth Observation, Sanya, Hainan Province, P.R. China; Centro de Investigacion Cientifica y de Educacion Superior de Ensenada Division de Fisica Aplicada, MEXICO

## Abstract

From multiple raster datasets to spatial association patterns, the data-mining technique is divided into three subtasks, i.e., raster dataset pretreatment, mining algorithm design, and spatial pattern exploration from the mining results. Comparison with the former two subtasks reveals that the latter remains unresolved. Confronted with the interrelated marine environmental parameters, we propose a ***T***ree-based ***A***pproach for e***X***ploring ***M***arine ***S***patial ***P***atterns with multiple raster datasets called TAXMarSP, which includes two models. One is the ***T***ree-based ***C***ascading ***O***rganization ***M***odel (TCOM), and the other is the ***S***patial ***N***eighborhood-based ***CA***lculation ***M***odel (SNCAM). TCOM designs the “Spatial node→Pattern node” from top to bottom layers to store the table-formatted frequent patterns. Together with TCOM, SNCAM considers the spatial neighborhood contributions to calculate the pattern-matching degree between the specified marine parameters and the table-formatted frequent patterns and then explores the marine spatial patterns. Using the prevalent quantification Apriori algorithm and a real remote sensing dataset from January 1998 to December 2014, a successful application of TAXMarSP to marine spatial patterns in the Pacific Ocean is described, and the obtained marine spatial patterns present not only the well-known but also new patterns to Earth scientists.

## 1. Introduction

Marine spatial pattern represents abnormal variations in one to several marine environmental parameters, e.g., sea-surface temperature (SST), sea-surface chlorophyll-a (Chl-*a*), sea-surface precipitation (SSP), and sea level anomaly (SLA), that occur or co-occur in a specified spatial region. Marine spatial patterns have become a hot issue in global climate changes [1] and play an important role in finding a regional essential climate variable [2,3]. An abnormal variation means a variation relative to an averaged status during a specified long-term series, e.g., monthly, seasonal, and annual abnormal variations. Long-term remote sensing images constitute the main source of continuous and consistent information about Earth’s land and oceans and offer new opportunities to improve our understanding of these marine spatial patterns on a large scale [4,5]. As an inductive method, spatiotemporal data mining shows more promise for discovering spatial patterns among multiple geographic parameters than the traditional statistical analysis [6–8], especially with the remote sensing images in recent decades [3,9,10].

Frequent pattern mining is a promising step to generate meaningful association knowledge, and this step accounts for most of the tasks in the mining process. Thus, the present study uses frequent pattern mining to replace the whole mining process to analyze the exploration from table-formatted patterns to spatial ones. From the raster datasets generated from remote sensing products to the marine spatial pattern generated from data mining, the whole mining process can be divided into three subtasks. The first task preprocesses the remote sensing images to construct the mining transaction table. The second task designs the mining algorithms to determine the table-formatted frequent patterns. The third task obtains marine spatial patterns from the table-formatted frequent patterns of all grid pixels. Regarding the first and second subtasks, many technologies were developed in the past few decades through extensive studies on their frameworks [3,11–14] and algorithms [15–17]. However, insufficient work has been done on the exploration of the spatial association patterns resulting from raster datasets. Therefore, a large opportunity is open to design more efficient strategies to obtain the spatial association patterns compared with the image pretreatment and mining algorithm [18].

To obtain the marine spatial patterns from table-formatted frequent patterns, an efficient structure is needed for storing and representing these table-formatted patterns. The present work intends to enhance this study. To date, traditional methods that deal with these patterns have been roughly divided into several types: textual descriptions and table-based views, scatter and parallel coordinate plots [19,20], mosaic and its variants [21], matrix representation [22], and graph-based views [23]. These techniques visualize all mined frequent patterns in a single view and struggle to deal with complex data and large collections of frequent patterns [18]. In addition, such techniques have only focused on a single-grid pixel and did not consider geospatial relationships. For this purpose, Bertolotto et al. (2007) and Compieta et al. (2007) integrated components from Google Earth and Java3D to visualize data, geographical parameters, and association patterns with multiple panels, i.e., antecedent, consequent, association-rule-extraction, and other panels [24,25].

Actually, the frequent patterns that arise from remote sensing datasets are complicated, i.e., each grid pixel may have several patterns, and each pattern may involve several geographical parameters. These complicated patterns require sophisticated organization model. Our previous work designed an interactive framework with three complementary components, namely, three-dimensional pie charts, two-dimensional variation maps, and triple-layer mosaics, to visualize marine association patterns [26]. Because only a few geographical parameters were involved in the data-mining model, implementing the three complementary visualization components was easy. Once the association patterns involve a large number of geographical parameters, vividly and intuitively visualizing many groups of triple-layer mosaics in the triple-layer mosaic component will not be very easy. In the recursive “Dimension–Attributes” visualization framework [11], a group of spatial thematic maps were used to display the association patterns with multiple marine parameters. Because only the association patterns with maximum confidence are considered, the other association patterns in the same grid pixel are lost.

Previous studies were not effective in extracting frequent patterns from sensing images that have multiple patterns in a pixel. To resolve the grid pixel with both several frequent patterns and multiple marine parameters, we propose a novel ***T***ree-based ***A***pproach for e***X***ploring ***M***arine ***S***patial ***P***atterns with multiple raster datasets called TAXMarSP. TAXMarSP consists of two models to effectively extract frequent patterns from sensing images with multiple patterns in one pixel. One is the ***T***ree-based ***C***ascading ***O***rganization ***M***odel (TCOM), which stores the table-formatted frequent patterns, and the other is the ***S***patial ***N***eighborhood-based ***CA***lculation ***M***odel (SNCAM), which explores marine spatial patterns from table-formatted ones by calculating the pattern matching degree between the specified marine parameters and frequent patterns. The remainder of this paper is organized as follows. Section 2 discusses the scientific problems of exploring marine spatial patterns from table-formatted frequent patterns resulting from multiple raster datasets and then proposes an analysis framework for resolving such problems. Section 3 presents the TCOM for storing table-formatted frequent patterns, and Section 4 presents the SNCAM for exploring marine spatial patterns from table-formatted patterns by calculating the pattern match degrees. A case study on exploring marine spatial patterns in the Pacific Ocean is described in Section 5, whereas Section 6 presents our discussion and conclusions.

## 2. Framework for exploring marine spatial patterns from the table-formatted frequent patterns mined with raster datasets

### 2.1. Problems

In a raster format, each grid pixel has several frequent patterns that link the marine environmental parameters. Each frequent pattern in a specified grid pixel involves several marine parameters, and each of them possesses quantification levels, which represent their variation degrees. In other words, each grid pixel has three meanings, namely, pattern, parameter, and variation information.

**Fig 1:**
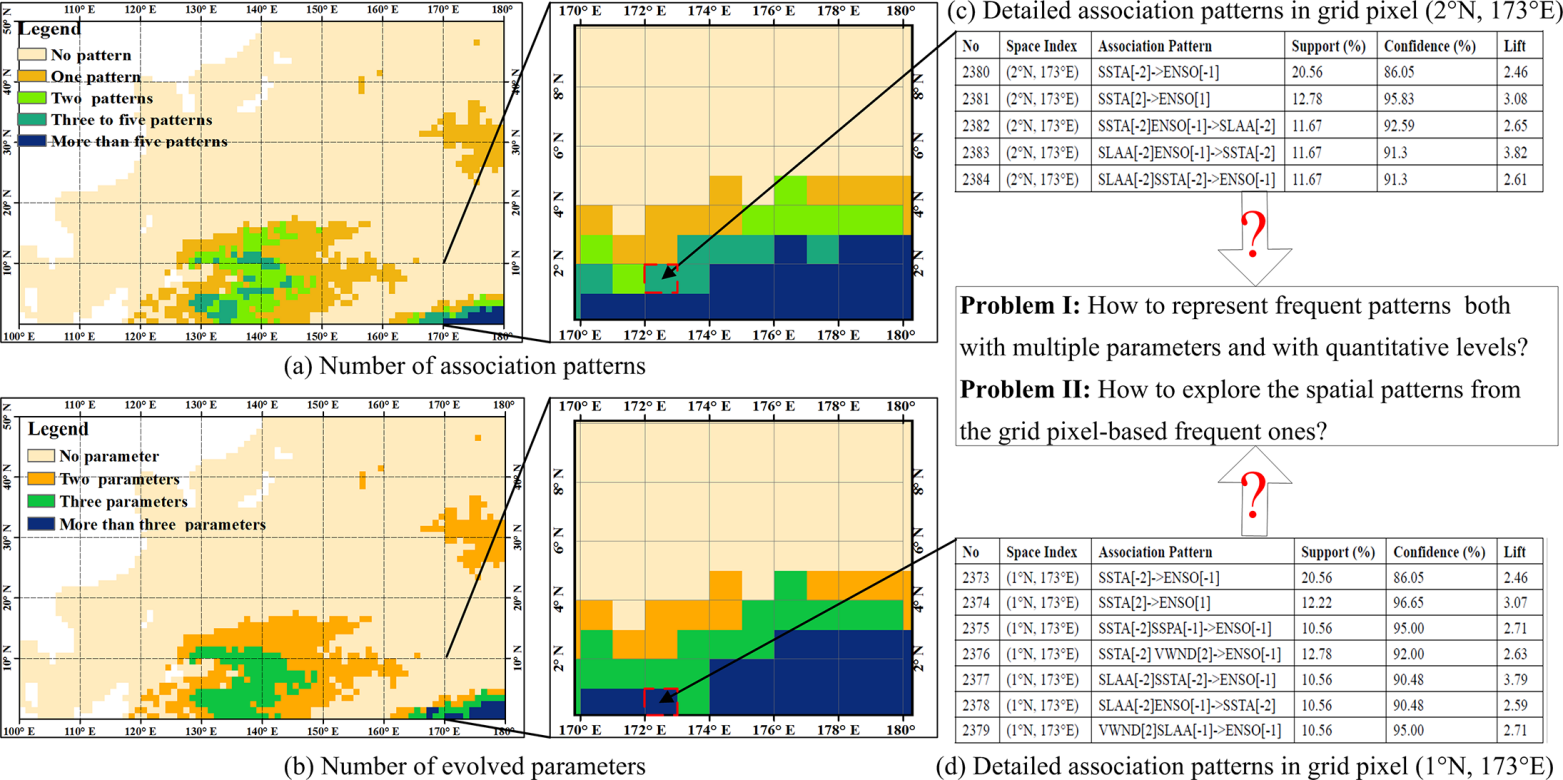
Description of the association patterns resulting from raster datasets.

Fig 1 shows the problems of exploring marine association patterns resulting from remote sensing images among multiple marine parameters, and each marine parameter is ranked into five quantification levels. The mining algorithm is based on the MIQarma [17], and the marine environmental parameters include monthly SST anomaly (SSTA), Chl-*a* anomaly (CHLA), SSP anomaly (SSPA), SLA anomaly (SLAA), U-component sea-surface wind, V-component sea-surface wind, and one of the signals of global change, i.e., the El Niño Southern Oscillation (ENSO) phenomenon. The five levels are -2, -1, 0, +1, and +2, indicating severe negative, slight negative, zero, slight positive, and severe positive changes, respectively. Fig 1(A) and 1(B) show the number of association patterns and the number of involved marine parameters in the northwestern Pacific Ocean. Fig 1(C) and 1(D) show the detailed association patterns in the specified grid pixels, i.e. (1°N,173°E) and (2°N,173°E). Fig 1 shows that in the equator region, the number of association patterns is more than five, and the involved parameters are not less than three. Furthermore, most of the association patterns in the adjacent grid pixels are similar. Thus, two challenges exist for exploring the marine spatial patterns from the raster datasets. One is to retrieve any frequent pattern with each parameter and with each level at the grid pixel locations. The other challenge is to explore the spatial patterns from the table-formatted frequent ones.

### 2.2. Exploration framework for spatial frequent pattern

**Fig 2:**
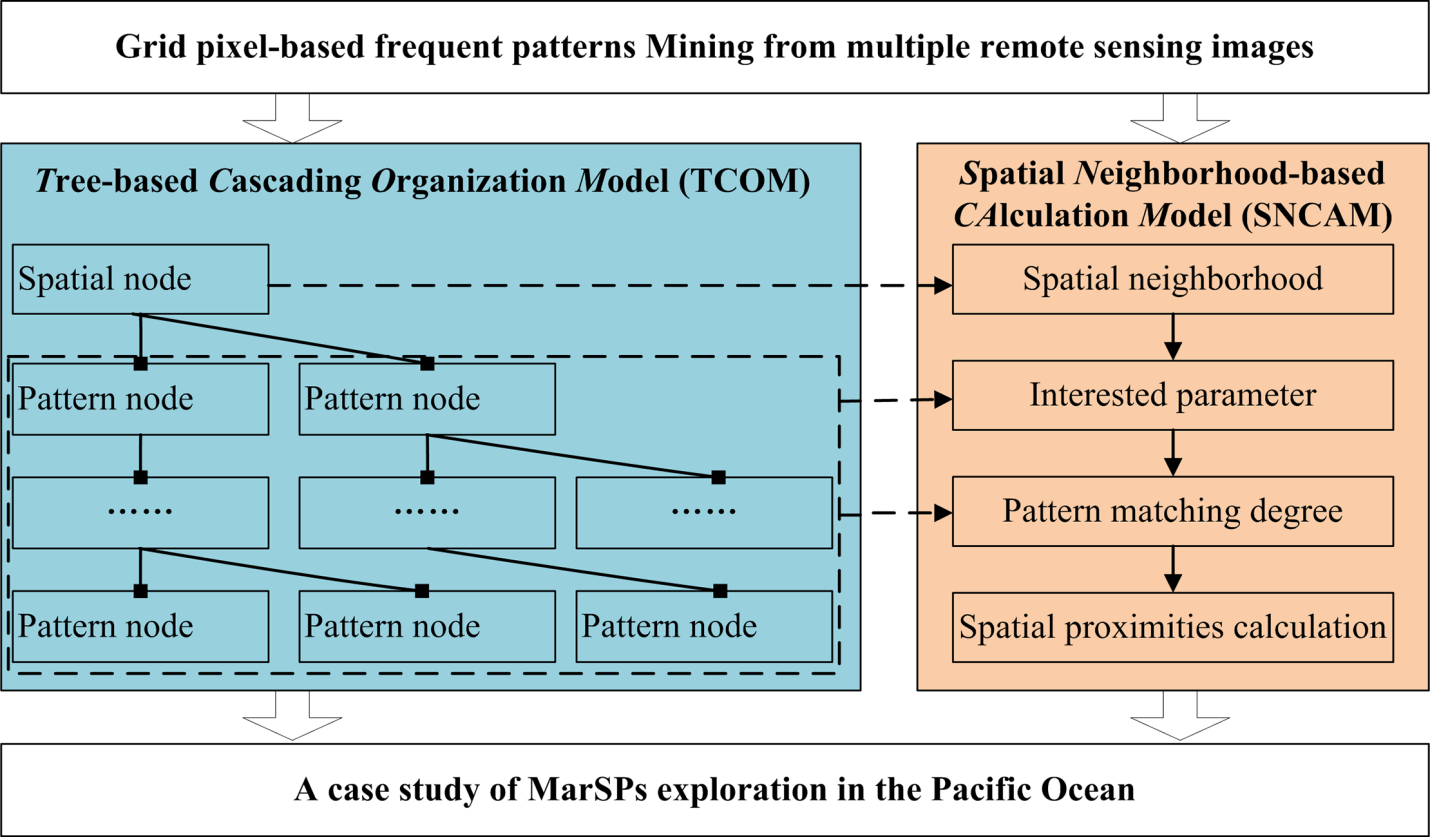
Framework of exploring marine spatial patterns from table-formatted frequent patterns.

For the first challenge, we need an organization model to simultaneously store the spatial location, parameters, and variation information. For the second one, we need a calculation model to deal with the similar patterns in the adjacent grid pixels. Thus, from the table-formatted frequent patterns to the marine spatial patterns with multiple raster datasets, this paper proposes an exploration framework, which includes four counterparts, i.e., input table-formatted frequent patterns, TCOM, SNCAM, and a case study in the Pacific Ocean. Fig 2 shows this exploration framework.

The input table-formatted frequent patterns are mined using existing algorithms, e.g., quantitative Apriori [27] and MIQarma [17]. After being satisfied with the user-specified thresholds, i.e., the minimum support and minimum confidence, such patterns are approved to be meaningful. TCOM designs the cascading structure with “Spatial node→Pattern node” to store the table-formatted frequent patterns. This structure not only helps retrieve the information of space, parameters, and variation in grid pixels but also supports SNCAM. SNCAM explores the marine spatial patterns by considering the contributions of the spatial neighborhoods. Meanwhile, the case study of marine spatial patterns in the Pacific Ocean proves the effectiveness and efficiency of our proposed framework.

## 3. TCOM

**Fig 3:**
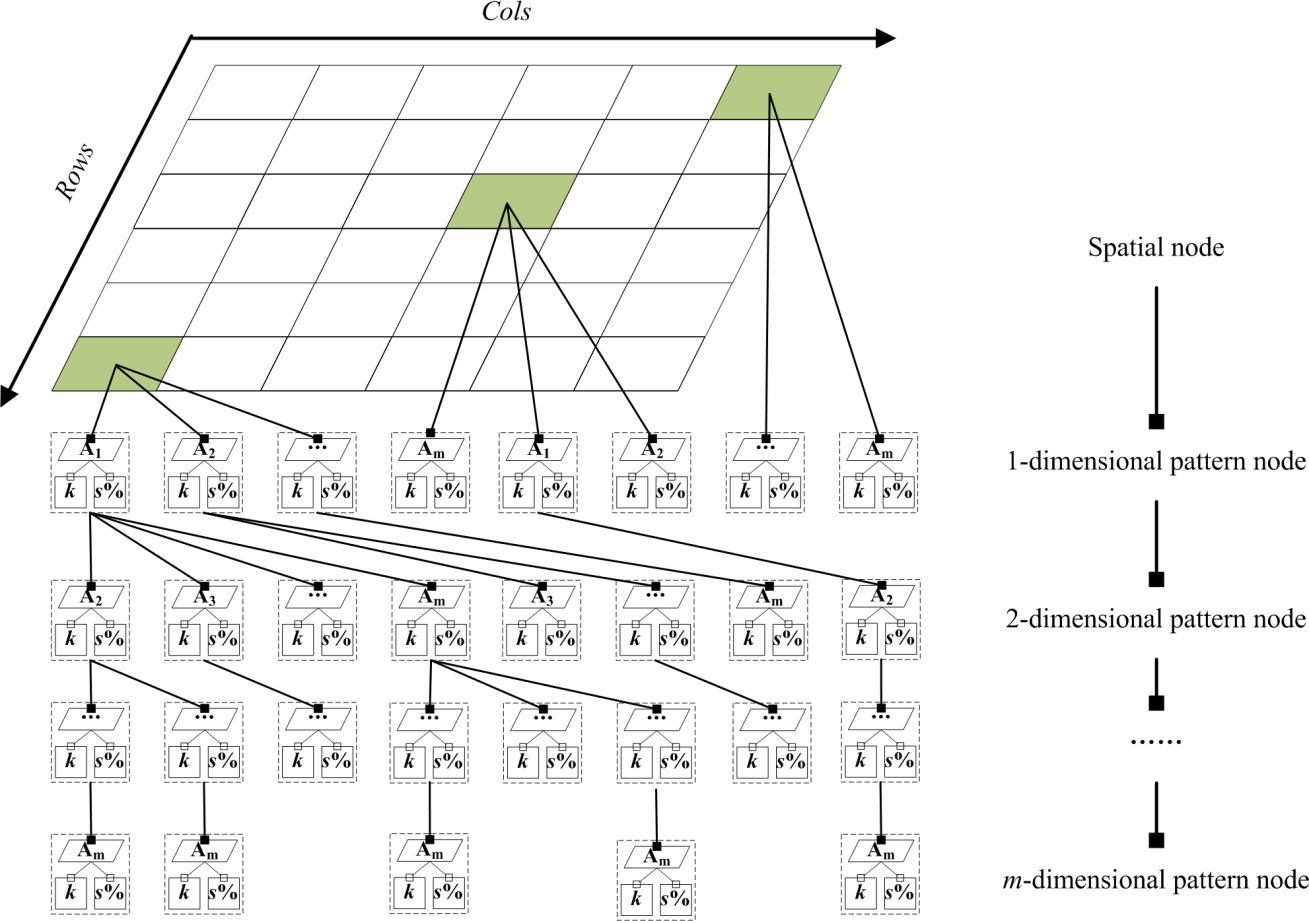
Structure of TCOM.

Because a grid pixel is uniform in representing spatial information, TCOM considers it as a root node, the one-dimensional frequent patterns as the second-layer node, the two-dimensional frequent patterns as the third-layer nodes, and so on. All layer nodes are denoted as pattern nodes. A TCOM with “Spatial node→Pattern node” is shown in Fig 3.

The spatial node stores the spatial information of frequent patterns in a form of (*row*, *col*), which has one-to-one correspondence with the row and column of the remote sensing image. Each spatial node has zero to *m* pattern nodes sorted in alphabetical order from left to right when *m* is not larger than the number of evolved marine parameters. Each pattern node has two leaves. From left to right, the two leaves store the variation type that represents the variation degree and a support that represents the occurrence probability of this frequent pattern. According to the frequent *m* patterns, the pattern nodes are classified into *m* layers. From top to bottom, one- to *m*-dimensional pattern nodes exist. The one-dimensional pattern node has zero to *m* pattern nodes, the two-dimensional pattern node has zero to *m*-1 pattern nodes, and so on. In this structure, we can easily obtain the spatial information and parameters of the frequent patterns.

Given a specified spatial location (*row*, *col*), the detailed steps to construct the pattern node are described as follows:

**Step 1:** Construct the one-dimensional pattern nodes

For all one-dimensional frequent patterns, extract their parameters, variation types, and supports. Sort their parameters in an increasing alphabetical order, and store them from left to right as one-dimensional pattern nodes. For each pattern node, first, determine its parameter and then the corresponding variation type and support. Finally store them from left to right as node leaves.

**Step 2:** Construct the (*m* + 1)-dimensional pattern nodes from the *m*-dimensional ones (where *m* is not less than one).

The pseudo-codes are based on one property, i.e., antimonotonicity, which means that all nonempty subsets of a frequent pattern must also be frequent, as proven in Ref. [28]. The construction process is described in Algorithm 1 with the pseudo-codes.

**Algorithm 1**. **An algorithm of constructing tree nodes**

    **Algorithm name**: ConstructingTreeNodesAlgorithm

    **Algorithm description**: Construct the (*m*+1)-dimensional pattern nodes from 2 *m*-dimensional ones (*m* is not less than one).

    **Input parameters**: *m*-dimensional pattern nodes, i.e., *m*-*N*, frequent (*m*+1) patterns, i.e., (*m*+1)-*F*.

    **Output parameters**: (*m*+1)-dimensional pattern nodes i.e., (*m*+1)-*N*.

    ***ConstructingTreeNodesAlgorithm***(*m*-*N)*, (*m*+1)-*F*, (*m*+1)-*N*)

    **FOR** each frequent (*m*+1)-pattern (*m*+1)-*f*, (*m*+1)-*f ∈*(*m*+1)-*F*

        **Extract** its parameters and reorganize them into a set in the form (*A*_*1*_[*k*_*1*_]*A*_2_[*k*_*2*_]*…A*_*m*_[*k*_*m*_]*A*_*m+1*_[*k*_*m+1*_]), which is sorted in an increasing alphabetical order

        **Extract** the nodes from left to right side one by one at the *m*-dimensional pattern node layer, denoted as (*Node*_1_, *Node*_2_, *…*, *Node*_N_), *NODE*_N_ is the total number of frequent *m*-patterns

        **FOR** the *i*th node in *NODE*, denoted as *i*th-*Node*, *i*th-*Node∈ NODE*, where *i* is not less than one and not greater than *N*

            **Find** the parent nodes of *i*th-*Node* step by step from (*m-*1)-dimensional pattern node layer to the one-dimensional node layer and reorganize them into a set in the form of (*Node*_1_ *Node*_2_*… Node*_m_)

            **IF** (*Node*_1_*Node*_2_*… Node*_m_) is a subset of (*A*_*1*_[*k*_*1*_]*A*_2_[*k*_*2*_]*…A*_*m*_[*k*_*m*_] *A*_*m+1*_[*k*_*m+1*_])

                **Calculate** their difference set, one item, denoted as *Node*, by *Node* = (*A*_*1*_[*k*_*1*_]*A*_2_[*k*_*2*_]*…A*_*m*_[*k*_*m*_]*A*_*m+1*_[*k*_*m+1*_]) -(*Node*_1_*Node*_2_*… Node*_m_)

                *Node* is taken as a new node, *Node*_m+1_, at the *m*-dimensional pattern node layer, and the tree (*Node*_1_→*Node*_2_→*…*→*Node*_m_) is appended, forming a new tree with (*Node*_1_→*Node*_2_→*…*→*Node*_m_*→Node*_m+1_)

                **Update** the tree

                **Break**

            **ELSE**

                *i* = *i*+1

            **END IF**

        **END FOR**

    **END FOR**

**Line 182** is a discriminant criterion to determine where to add a new node. If it is true, a new node is appended and forms a new tree with (*Node*_*1*_→*Node*_*2*_→…→*Node*_*m*_→*Node*_*m+1*_) (**Lines 183**–**188**). Then, the next frequent (*m*+1) pattern is completed (go to **Line 172**). If not, the process goes to **Line 191**, and the next node in *NODE* will be completed. From **Lines 177** to **193**, a loop is completed until the frequent (*m*+1) patterns are appended into the tree. **Lines 172**–**194** are repeated to go through all frequent (*m*+1) patterns.

To clearly show the process of constructing the TCOM, we provide an example based on the simulated data.

**Example 1**: We provide a specified spatial location (row, col), which has six marine parameters (A_1_,A_2_,…,A_6_) with quantitative changes during a time series of 10 timestamps. The quantitative data are listed in Table 1. The +1, 0, and −1 marine parameters mean positive, zero, and negative changes, respectively.

**Table 1:** Quantitative data in the database for Example 1.

	A_1_	A_2_	A_3_	A_4_	A_5_	A_6_
0	+1	-1	+1	0	-1	+1
1	-1	0	+1	0	+1	+1
2	-1	+1	0	-1	0	0
3	+1	-1	+1	-1	+1	0
4	-1	-1	+1	0	0	+1
5	+1	-1	-1	+1	+1	-1
6	+1	0	+1	0	+1	-1
7	0	-1	-1	-1	0	0
8	+1	-1	0	0	+1	0
9	0	-1	+1	-1	0	-1

**Fig 4:**
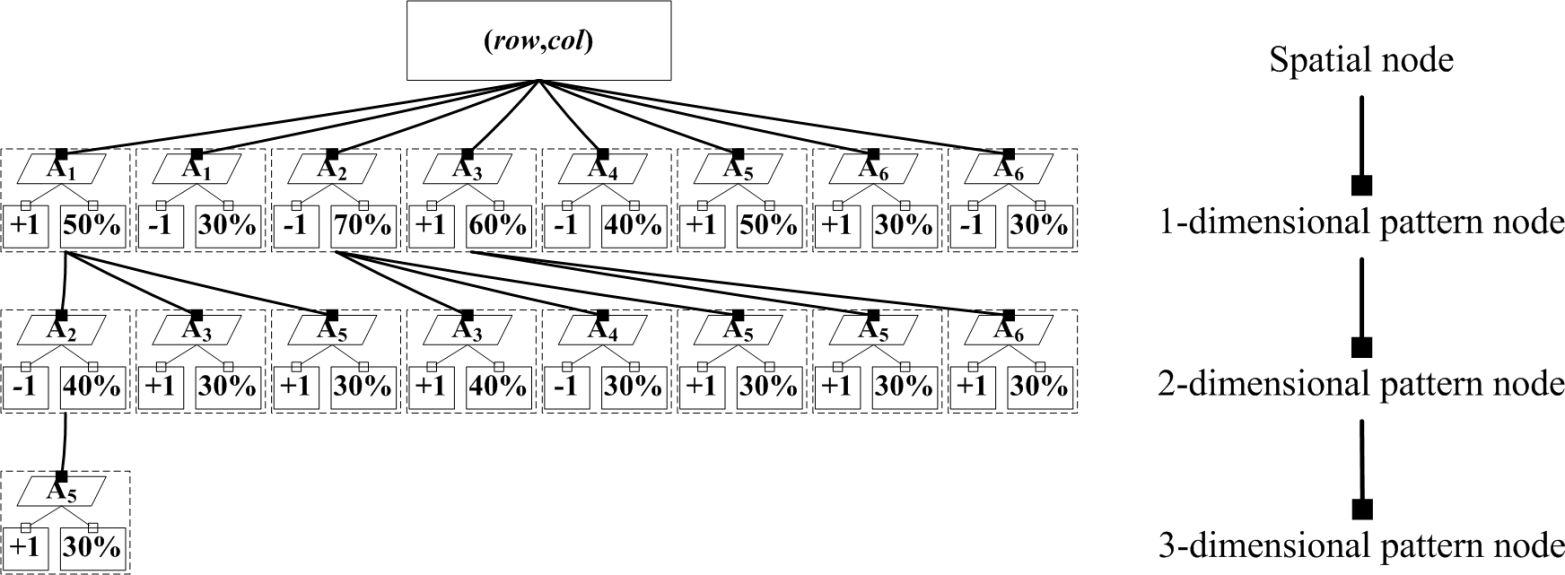
Tree-based cascading organization of Example 1.

To simplify the process flow, the support threshold is set to 30%, and the frequent patterns are listed in Tables 2–4. According to the Algorithm 1, the frequent pattern tree is shown in Fig 4, and the detailed steps in constructing this tree are described as follows:

**Step 1:** Take the spatial node (*row*, *col*) as a root node.

**Step 2:** According to the number of frequent one-dimensional patterns in Table 2, design eight pattern nodes, which store parameter names A_1_, A_1_, A_2_, A_3_, A_4_, A_5_, A_6_, and A_6_ from left to right.

**Step 3:** For each pattern node, design its two leaves, which store the parameter variation type and support from left to right.

**Step 4**: Organize the one-dimensional pattern nodes into a new set from left to right and denote as *NODE*, i.e., A_1_[+1]A_1_[–1]A_2_[–1]A_3_[+1]A_4_[–1]A_5_[+1]A_6_[+1]A_6_[–1]. Within *NODE*, a parameter and its variation type form its element, i.e., node. Eight nodes exist.

**Step 5:** For each frequent two-dimensional pattern listed in Table 3, extract its parameters and its variation types, reorganize them into a new set in an increasing alphabetical order, and denote them as *AppendingPattern*, e.g., the first frequent two-dimensional pattern is A_1_[+1]A_2_[–1].

**Step 6:** For each node in *NODE*, find its parent pattern nodes one by one from the top to the bottom layers and reorganize these nodes into a new set, denoted as *RawPattern*. Because the one-dimensional pattern node has no parent nodes, the new set represents itself, e.g., the new set of the first node in *NODE* is A_1_[+1].

**Step 7:** Go through all *RawPatterns* until *RawPattern* is found, which is a subset of *AppendingPattern*. Then, calculate their difference set, which is one parameter, e.g., the difference set between the first frequent two-dimensional pattern and the first node in *NODE* is A_2_[–1], denoted as a new node. Append *RawPattern* to the new node and form a new tree, i.e., A_1_[+1] → A_2_[–1]. Then, update it.

**Step 8:** Repeat Steps 5 to 7 until all frequent two-dimensional patterns are appended.

**Step 9:** Perform similar operation as in Step 4 to reorganize the two-dimensional pattern nodes into a new *NODE*, i.e., A_2_[–1] A_3_[+1] A_5_[+1] A_3_[+1] A_4_[–1] A_5_[+1] A_5_[+1]A_6_[+1].

**Step 10:** Perform similar operations as in Steps 5 to 8 to construct the three-dimensional pattern nodes.

**Table 2:** Frequent one-dimensional patterns from Table 1.

	Pattern	Support (%)
0	A_**1**_[+1]	50
1	A_**1**_[–1]	30
2	A_**2**_[–1]	70
3	A_**3**_[+1]	60
4	A_**4**_[–1]	40
5	A_**5**_[+1]	50
6	A_**6**_[+1]	30
7	A_**6**_[–1]	30

**Table 3:** Frequent two-dimensional patterns from Table 1.

	Pattern	Support (%)
0	A_**1**_[+1]A_**2**_[–1]	40
1	A_**1**_[+1]A_**3**_[+1]	30
2	A_**1**_[+1]A_**5**_[+1]	30
3	A_**2**_[–1]A_**3**_[+1]	40
4	A_**2**_[–1]A_**4**_[–1]	30
5	A_**2**_[–1]A_**5**_[+1]	30
6	A_**3**_[+1]A_**5**_[+1]	30
7	A_**3**_[+1]A_**6**_[+1]	30

**Table 4:** Frequent two-dimensional pattern from Table 1.

	Pattern	Support (%)
0	A_**1**_[+1]A_**2**_[–1]A_**5**_[+1]	30

Among the above steps, Steps 1–3 construct the one-dimensional pattern nodes, Steps 4–8 construct the two-dimensional ones, and Steps 9 and 10 construct the three-dimensional ones.

## 4. SNCAM

According to Tobler’s First Law of Geography, all frequent patterns on a geographic surface are related to one another, but the closer patterns are more strongly related than the more distant ones [29]. In other words, frequent pattern mining from raster datasets tends to appear in spatial clusters. Thus, we design SNCAM to explore the spatial pattern.

Considering the challenges associated with simultaneously analyzing complicated frequent patterns at the same location, first, we determine which parameters are of interest. Then, we transform such patterns into a series of frequent patterns with the given parameters. Finally, we use the spatial thematic map to represent them. The choice of which parameters to analyze depends on the interests of the user.

**Fig 5:**
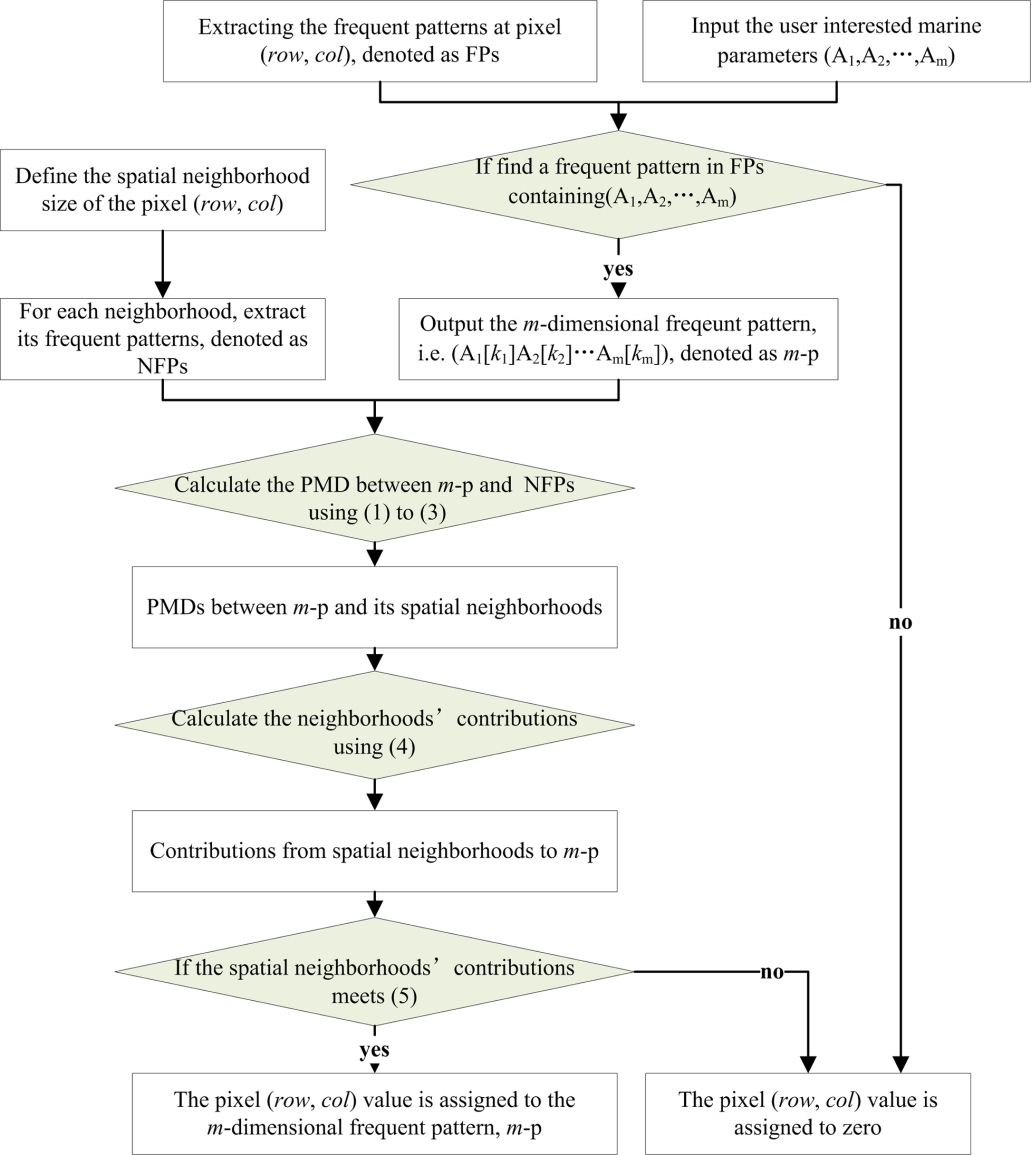
Workflow of SNCAM.

To calculate the spatial ***P***attern ***M***atching ***D***egree (PMD), SNCAM embeds the contributions of the spatial neighborhoods. Fig 5 shows the workflow of SNCAM for a given spatial location, (*row*, *col*) and the marine parameters of interests.

In Fig 5, the frequent patterns at pixel (*row*, *col*) are denoted as FPs, whereas those of the spatial neighborhoods are denoted as NFPs. Given (A_1_,A_2_,…, A_m_) as the user frequent patterns of interest at the *m*-dimensional pattern node, if the FPs at (*row*, *col*) do not contain (A_1_,A_2_,…, A_m_), the pixel (*row*, *col*) value is assigned a value of zero. If the FPs contain (A_1_,A_2_,…, A_m_), the *m*-dimensional frequent pattern (A_1_[k_1_]A_2_[k_2_]… A_m_[k_m_]) is denoted as *m-p*, and the following steps should be carried out to assign the pixel (*row*, *col*) value.

**Step 1:** Determine the spatial neighborhood window size of the pixel (*row*, *col*) in both directions, known as *w*. For each neighborhood, calculate its PMD to *m-p* using Eqs (1) to (3). Eq (3) is a recursive function.

$$\eta_{PMD}(i,j)<m-p,\text{NFP}s>=\frac{\sum_{k=1}^{K}\eta_{PMD}(i,j)<m-p,\text{NFP}s[k]>}{K}$$ (1)

$$\eta_{PMD}(i,j)<m-p,\text{FAP}s[k]>=\{\begin{array}{ccc} 1.0 & & m-p\in \text{NFP}s[k]\\ -1.0 & & m-p'\in \text{NFP}s[k]\\ \frac{\sum_{s=1}^{s=m}f(s^{th}(m-1)-p)}{m} & & \text{others} \end{array},$$ (2)

$$f(s^{th}(m-r)-p)=\{\begin{array}{llll} 1.0 & & & \left(m-r)-p\in \text{NFP}s[k\right]\\ -1.0 & & & \left(m-r)-p'\in \text{NFP}s[k\right]\\ 0 & & & (m-r)-p\notin \text{NFP}s[k]\&m-r=1\\ \frac{\sum_{s=1}^{s=m-r}f(s^{th}(m-r-1)-p)}{m-r} & & & (m-r)-p\notin \text{NFP}s[k]\&m-r\neq 1 \end{array},$$ (3)

where NFPs represent the frequent pattern of a spatial neighborhood at pixel (*i*,*j*) and *i* and *j* represent the row and column, respectively, in a spatial neighborhood. NFPs[*k*] is the *k*th frequent pattern of NFPs, *K* is the total number of frequent patterns, *m−p* is the *m*-dimensional pattern to be matched, *m−p*' is the anti-pattern of *m−p*, and (*m−r*)−*p* is a one (*m-r*)-dimensional sub-pattern of *m−p*. Considering pixel (*row*,*col*) as the center and *row*−*w*/2 ≤ *i* ≤ *row* + *w*/2 and *col*−*w*/2 ≤ *j* ≤ *col* + *w*/2, we learn that *m*−*p* ∈ NFP*s*[*k*] means that *m−p* belongs to NFP*s*[*k*], and *m*−*p*' ∈ NFP*s*[*k*] means that the anti-pattern of *m−p* belongs to NFP*s*[*k*].

**Example 2:** Given pixel (row, col), one of its matched frequent pattern is A_1_[+1]A_2_[+1], denoted as m−p. The PMDs from its spatial neighborhoods with a 3 × 3 window size are listed in Table 5, where the frequent patterns of the spatial neighborhoods are denoted as NFPs.

**Table 5:** PMDs from the spatial neighborhoods to the central pixel.

	Spatial location	NFPs	*η*_*PMD*_	Description
1	(*row*− 1, *col*− 1)	A_1_[+1]A_2_[+1]	1.0	*m*−*p* ∈ NFP*s*
2	(*row*− 1, *col*)	A_1_[+1]A_2_[+1]A_3_[+1]	1.0	*m*−*p* ∈ NFP*s*
3	(*row*− 1, *col*+1)	A_1_[+1]	0.5	One subset of *m*−*p* belongs to NFPs
4	(*row*, *col*− 1)	A_2_[+1]	0.5	One subset of *m*−*p* belongs to NFPs
5	(*row*, *col*+1)	A_1_[+1]A_2_[−1]	0	One subset of *m*−*p* belongs to NFPs, and one anti-subset belongs to NFPs
6	(*row*+1, *col*− 1)	A_1_[−1]A_2_[−1]	−1.0	Anti-pattern of *m*−*p* belongs to NFPs
7	(*row*+1,*col*)	A_2_[−1]	−0.5	One anti-subset belongs to NFPs
8	(*row*+1, *col*+1)	A_1_[+1]A_2_[−1], A_1_[+1]A_3_[−1]	0.25	The matched degree of the first pattern is zero, and the second is 0.5. From Eq (1), the total matched degree is (0+0.5)/2.

**Step 2:** Calculate the neighborhood contributions to *m*−*p* using Eq (4) according to the spatial neighborhood PMDs.

$$C_{Ngh}(row,col)=\frac{\sum_{i=0}^{w-1}\sum_{j=0}^{w-1}\eta_{PMD}(i,j)-1}{w\times w-1}$$ (4)

**Step 3:** Assign the pixel (*row*, *col*) value according to the inequality in (5); if (5) is true, the pixel value is set to A_1_[k_1_]A_2_[k_1_]… A_m_[k_m_]. Otherwise, the value is zero.

$$C_{Ngh}>\tau_{c},$$ (5)

where *τ*_*c*_ is the contribution threshold.

## 5. Case study—Marine spatial patterns in the Pacific Ocean

**Fig 6:**
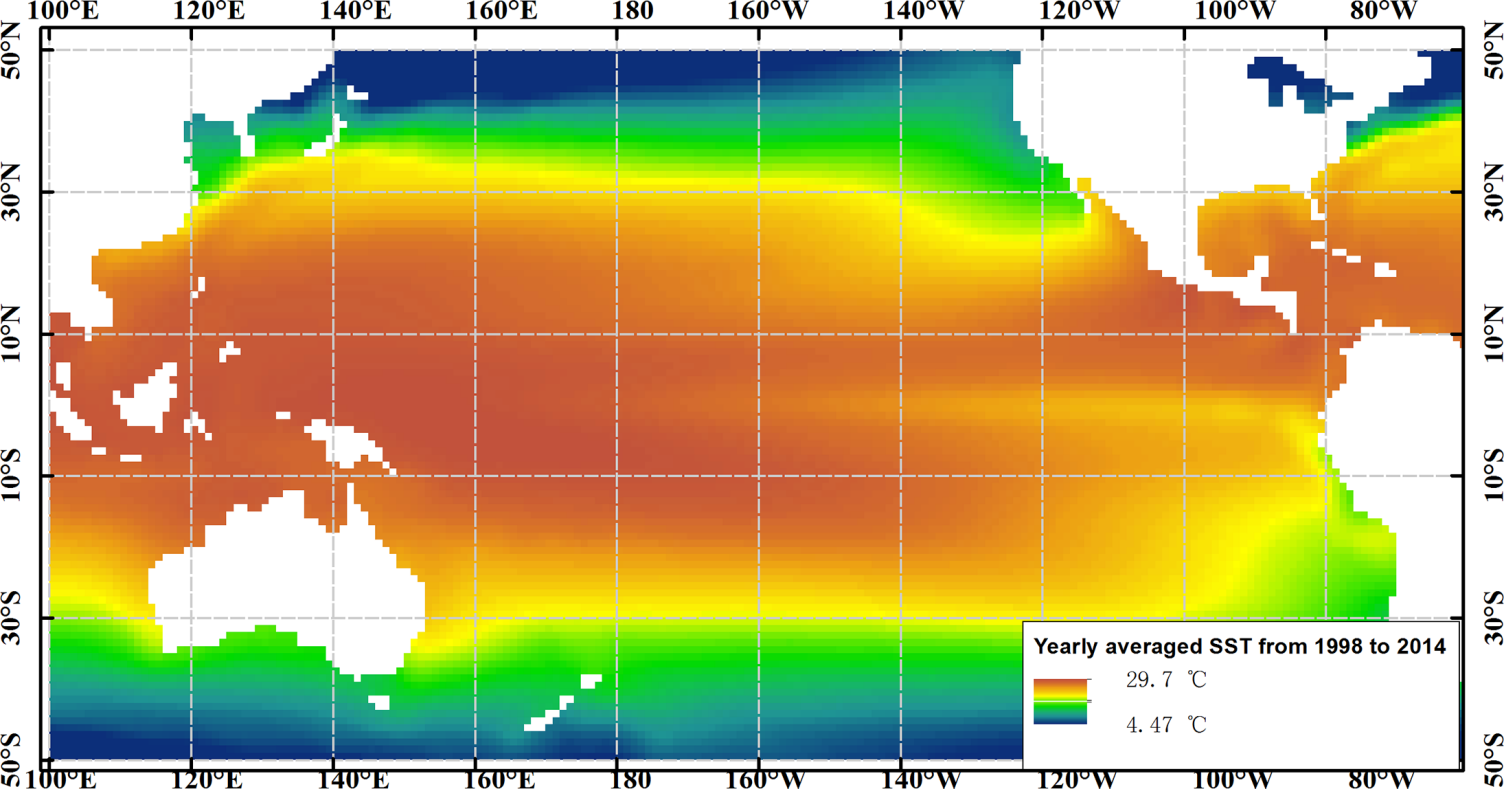
Research area. The background colors show the yearly averaged SST from 1998 to 2014.

Our study was conducted on long-term marine remote sensing products, including SST, Chl-*a*, SSP, and SLA. Multiple ENSO index (MEI) was used to identify the ENSO events. The Pacific Ocean from 100°E to 60°W and 50°S to 50°N, where it is sensitive to global climate change and regional sea–air interactions and is responsible for marine variations, was considered as a case study, as shown in Fig 6. Table 6 lists the summary of the used datasets. SST was obtained from (http://www.esrl.noaa.gov/psd/) and provided by NOAA/OAR/ESRL Physical Sciences Division [30]. Chl-*a* was obtained from the SeaWiFS and MODIS projects, including their level-3 standard mapped images [31]. SSP was obtained from Version 7 of the Tropical Rainfall Measuring Mission (TRMM Product 3B43), provided by the Goddard Distributed Active Archive Center (GES DISC DAAC). SLA was produced by Ssalto/Duacs and distributed by AVISO with the support of Cnes (http://www.aviso.oceanobs.com/duacs). The ENSO index was obtained from (http://www.esrl.noaa.gov/psd/enso/mei/) and provided by NOAA-ESRL Physical Sciences Division [32].

**Table 6:** Sources and resolutions of the raster datasets and MEI used in this study.

	Product	Source	Timespan	Temporal resolution	Spatial coverage	Spatial resolution
1	SST	NOAA/PSD	1981.12–2014.12	Monthly	Global	1° × 1°
2	Chl-*a*	SeaWiFS	1997.09–2010.11	Monthly	Global	9 × 9 km
		MODIS	2002.07–2014.12	Monthly	Global	9 × 9 km
3	SSP	TRMM	1998.01–2014.12	Monthly	Global	0.25° × 0.25°
4	SLA	AVISO	1993.01–2014.12	Monthly	Global	0.25° × 0.25°
5	ENSO	MEI	1950.01–2014.12	Monthly	-	-

### 5.1. Data pretreatment and frequent pattern discovery

To obtain uniform datasets from the raster datasets with the same spatial and temporal resolutions, the analysis period from January 1998 to December 2014 was selected. The monthly anomalies of the research area elements with a spatial resolution of 1° in the grid projection and with a time resolution of one month were calculated to remove the seasonal effects. The resulting anomalies were SSTA, SLAA, SSPA and CHLA, and the datasets are S1 Dataset, S2 Dataset, S3 Dataset and S4 Dataset, respectively. Thus, 100 × 200 grid pixels with 204 time series were quantified, yielding a total of 100 × 200 × 204 records with five parameters each (i.e., SSTA, CHLA, SSPA, SLAA, and MEI).

A combination of the mean and 1.0 standard deviation of the time series of each grid pixel was used to quantify the marine environmental parameters at each time interval into three levels. The value is defined as one when it is at a time interval greater than the mean plus 1.0 standard deviation. The value is defined as -1, when it is less than the mean less 1.0 standard deviation. The remaining value is defined as zero. The −1, 0, or +1 value indicates negative, zero, or positive change, respectively. MEI was quantified in the same manner, and −1, 0, and +1 indicate a La Niña event, neutral condition, and El Niño event, respectively. Using this algorithm, we have obtained ENSO events similar to those in Refs. [8], [33], and [34]. This is the core idea of the quantitative Apriori derived from the previous Apriori algorithm, which has been widely used in the data-mining domain. After many experiments and comparisons, the optimal support threshold was set to 10.0% [17], and the quantitative Apriori algorithm was used to discover the frequent patterns of each grid pixel one by one. The total number of mined frequent patterns is 14326, and S1 Table lists the frequent patterns of the grid pixel (0, 174°E) and its eight-neighborhood patterns. S1 Table lists too much information about the association patterns among two and more marine environmental parameters, and finding the spatial information where the specified marine environments interact as listed in S1 Table is very difficult, e.g., where the marine environments respond when a La Niña event occurs or where an abnormal increase in SSTA indicates the occurrence of a La Niña event, and so on. Thus, TCOM was used to store these table-formatted frequent patterns, whereas SNCAM was used to extract the spatial frequent patterns. In SNCAM, *τ*_*c*_ was set to zero, meaning that at least half of the neighborhoods contribute to the center pixel, i.e., the center pixel is ensured to be not an isolated noise.

### 5.2. Marine spatial patterns in the Pacific Ocean

With 10.0% support threshold, the marine spatial patterns indicate that the probability is not less than 10.0% when abnormal variations in one or several marine environmental parameters in a specified spatial region occur or co-occur. In other words, these abnormal variations in a specified spatial region last for at least 20.4 months. From the table-formatted patterns to the spatial patterns, spatial neighborhood window size *w* was set to 3 pixels (i.e., the latitude and longitude spatial ranges are 3°), and the spatial neighborhood contribution threshold was set to zero, meaning that at least half of the neighborhoods that contribute to the pattern must be matched.

**Fig 7:**
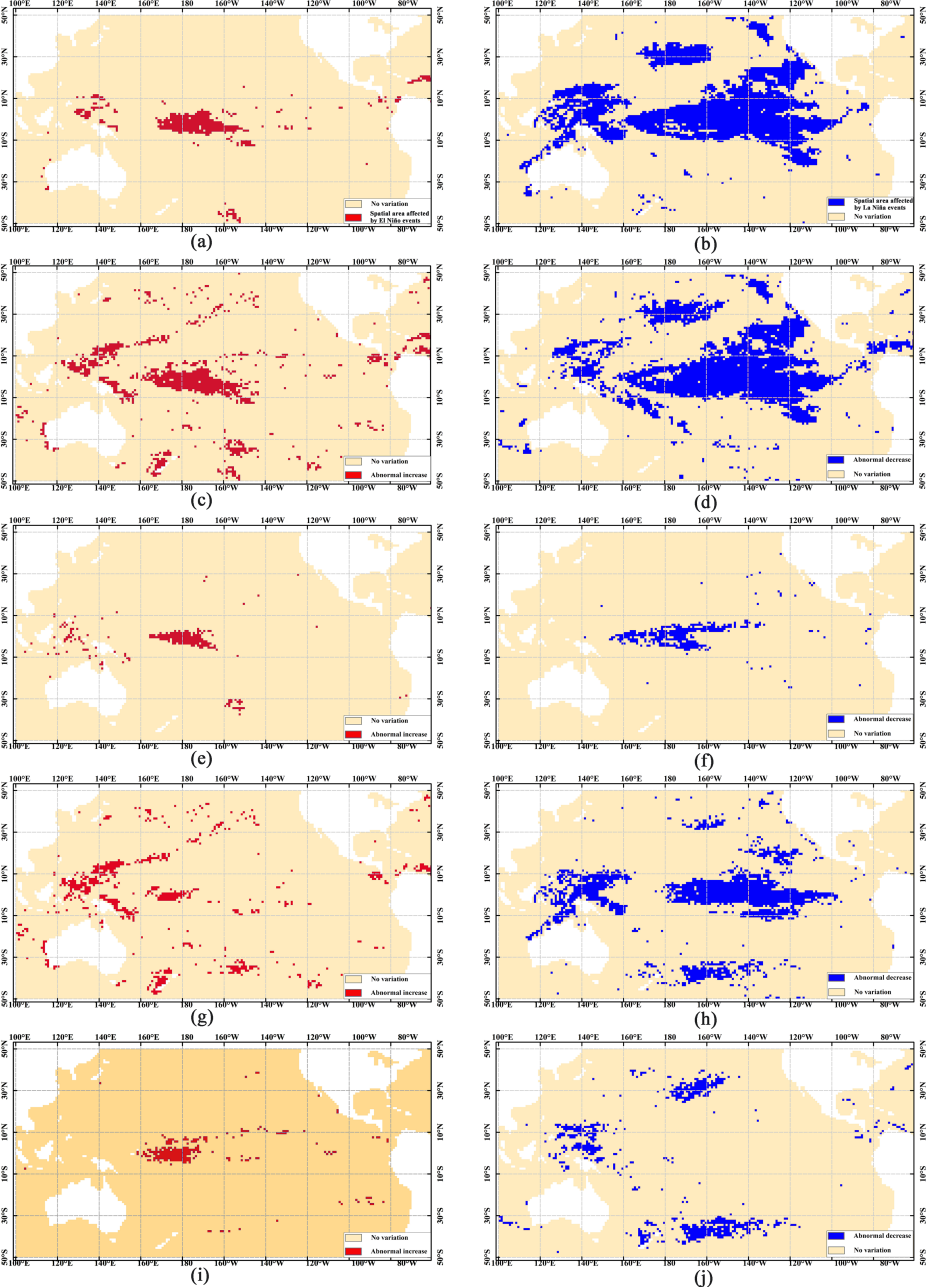
Spatial distribution of frequent one-dimensional patterns. (a) El Niño event. (b) La Niña event. (c) Abnormal increase in SSTA. (d) Abnormal decrease in SSTA. (e) Abnormal increase in SSPA. (f) Abnormal decrease in SSPA. (g) Abnormal increase in SLAA. (h) Abnormal decrease in SLAA. (i) Abnormal increase in CHLA. (j) Abnormal decrease in CHLA.

To illustrate the feasibility of our proposed method, a series of two-dimensional thematic maps was used to map the frequent spatial patterns. Because the same spatial region may have directly opposite characteristics, i.e., abnormal increase and decrease variations, from the table-formatted patterns to the spatial ones, the marine parameters of interest with a quantitative level should be given first. Using SNCAM, 10 frequent one-dimensional spatial patterns are obtained. They are El Niño/La Niña events (ENSO with +1/-1 level), SSTA abnormal increase/decrease (SSTA with +1/-1 level), SLAA abnormal increase/decrease (SLAA with +1/-1 level), SSPA abnormal increase/decrease (SSPA with +1/-1 level), and CHLA abnormal increase/decrease (CHLA with +1/-1 level), as shown in Fig 7.

**Fig 8:**
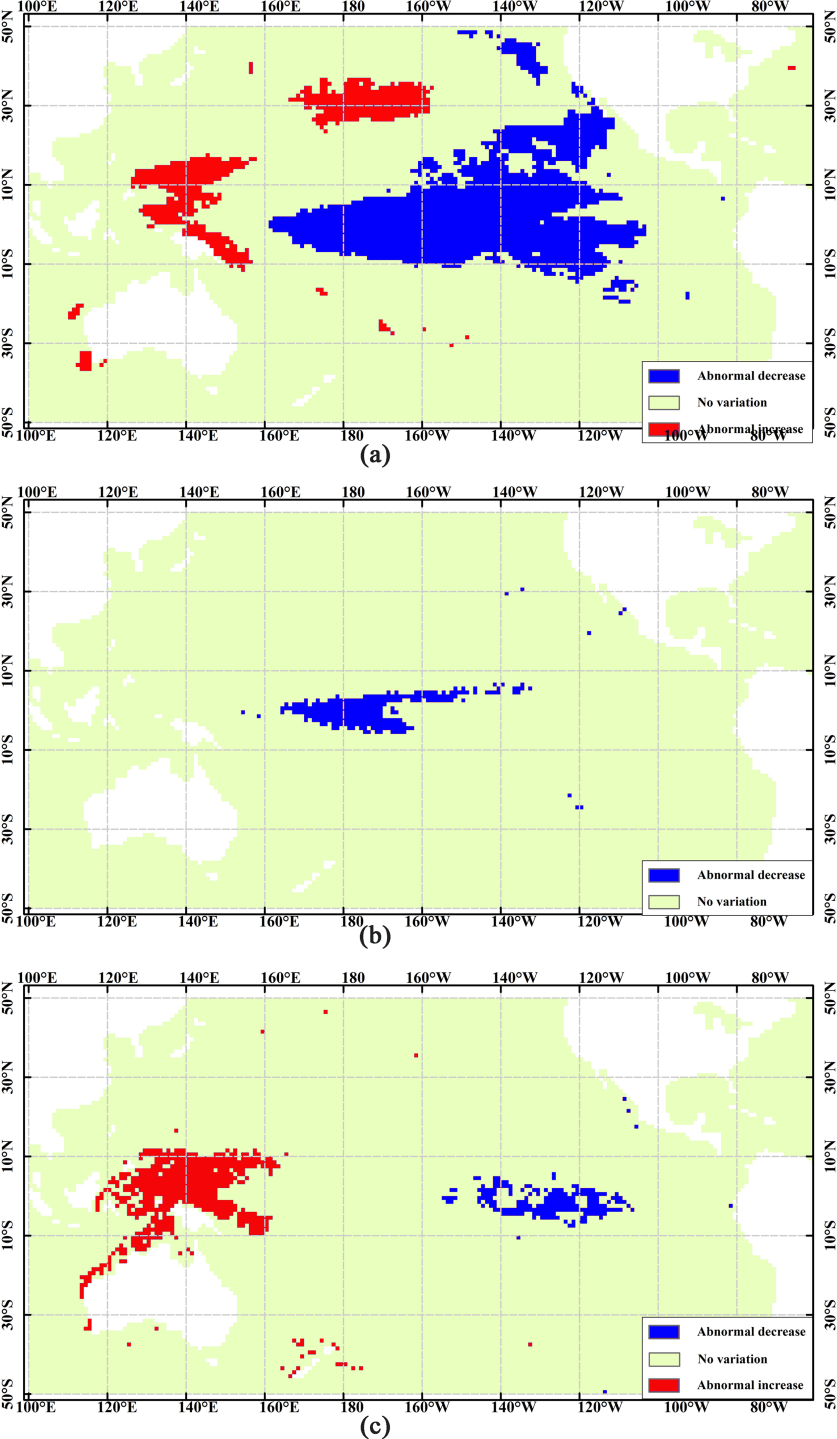
Spatial distribution of frequent two-dimensional patterns with La Niña events. (a) SSTA abnormal variations. (b) SSPA abnormal variations. (c) SLAA abnormal variations.

**Fig 9:**
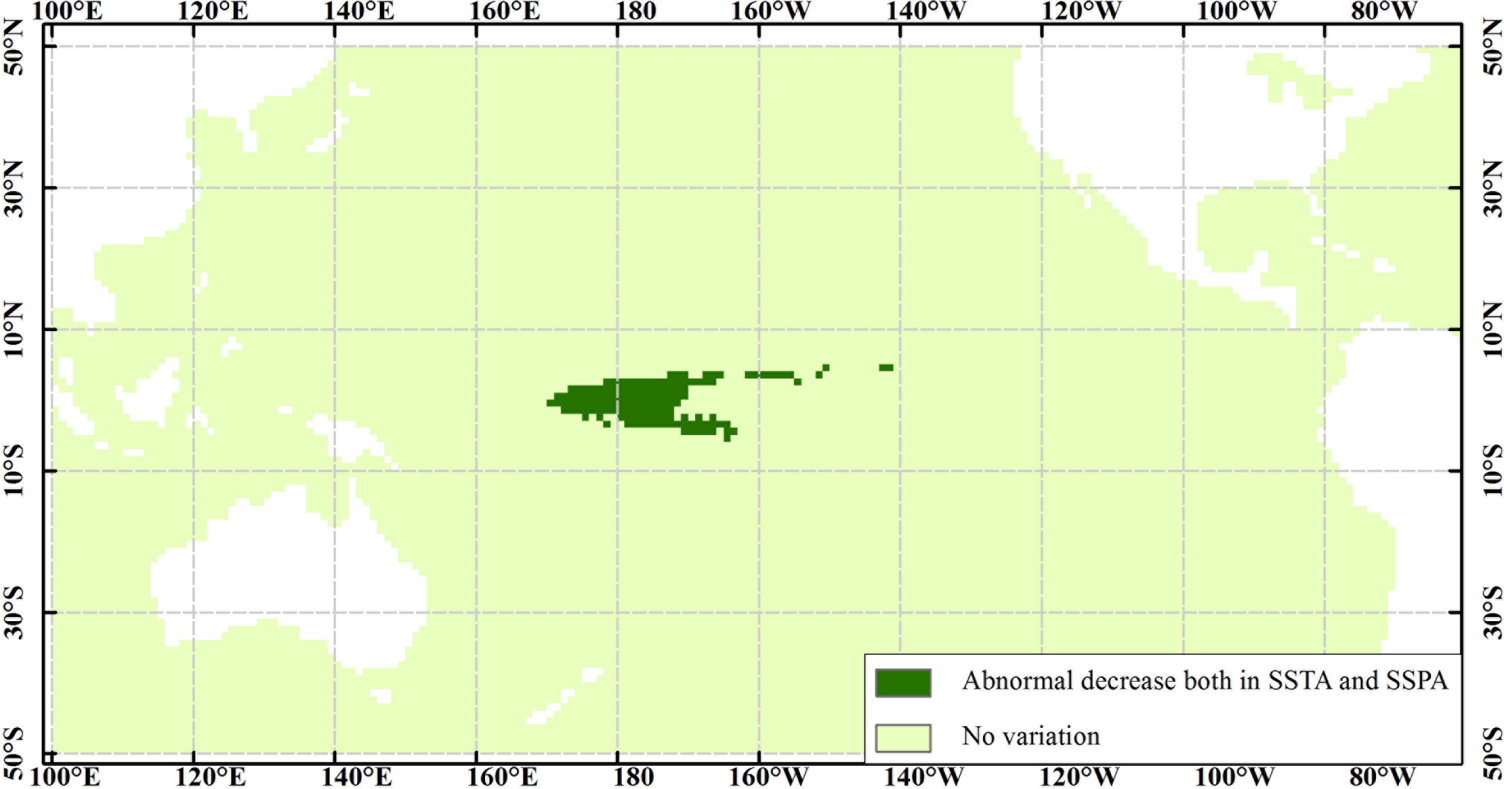
Spatial distribution among SSTA, SSPA, and a La Niña event.

ENSO is a dominant climate signal, which is a cycle of the alternating warm El Niño and cold La Niña. The relationships between ENSO and the marine environments comprise a very complicated and interrelated system [1]. Thus, we consider the La Niña event as a parameter of interest to obtain the marine spatial patterns with frequent two- and three-dimensional patterns. Based on SNCAM, we obtain three frequent two-dimensional spatial patterns. They are SSTA, SSPA, and SLAA during a La Niña event, as shown in Fig 8. In addition, we obtain one frequent three-dimensional spatial pattern among the SSTA, SSPA, and a La Niña event, as shown in Fig 9.

Fig 7 shows that directly opposite variations exist in the western and eastern Pacific Ocean. In other words, these regions are sensitive not only to El Niño and La Niña events but also to abnormal increase and decrease in the marine environmental parameters. In such regions, analyzing the spatial relationships using the traditional methods becomes challenging. Using TCOM and SNCAM, we can obtain the spatial relationship of one geographical parameter (Fig 7). We can also obtain the spatial patterns among several parameters (Figs 8 and 9). In addition, some of the obtained spatial patterns are well known to Earth scientists whereas others are not. For example, when La Niña events occur, the westward North Equatorial Current, South Equatorial Current, and eastward Equatorial Counter Current result in the decrease in the SSTA in the central and eastern Pacific Ocean and increase in the western Pacific Ocean, as shown in Fig 8(A). The increasing warm water in the western Pacific Ocean depresses the water mass transport, resulting in westward accumulation. Therefore, the SLA in the western Pacific Ocean increases, whereas that in the eastern Pacific Ocean decreases [Fig 8(C)]. Under the force of the trade winds and the Walker circulation, the rainfall shifts westward, and the SSPA in the middle of the tropical Pacific Ocean abnormally decreases [35] [Fig 8(B)]. However, further study is needed to determine the physical mechanisms behind the abnormal decrease in the SSTA along the California coast, the abnormal increase in the SSTA in the northern subtropical Pacific Ocean [Fig 8(A)], and the co-variations in the decrease in SSTA and SSPA (Fig 9).

## 6. Conclusions

To address the great challenges of dealing with table-formatted frequent patterns resulting from rule mining using multiple long-term raster datasets, we have proposed an original approach to explore marine spatial patterns named TAXMarSP. TAXMarSP includes two models, i.e., TCOM and SNCAM. TCOM stores the table-formatted frequent pattern and supports spatial information extraction, whereas SNCAM explores the spatial information from the pixel-based frequent patterns. A real dataset coming from multiple remote sensing products was used to explore marine spatial patterns in the Pacific Ocean. Among these marine spatial patterns, some are well known to Earth scientists, whereas the others are new patterns.

In summary, the main contributions of our algorithm and study are the following:

1. TAXMarSP linked the table-formatted frequent patterns to spatial information, which improved the capacities of dealing with multiple long-term raster datasets.
2. Using the “Spatial node→Pattern node,” TCOM simultaneously stored the spatial location, parameters, and variation degree of the frequent patterns. The spatial node layer helped obtain the spatial location, whereas the pattern node layers (from bottom to top) helped obtain any dimensional frequent patterns.
3. SNCAM considered the contributions from spatial neighborhoods when exploring the spatial patterns. Using spatial neighborhoods, the pseudo-frequent patterns were removed.
4. A case study within the Pacific Ocean using SSTA, SLAA, SSPA, CHLA, and MEI was conducted, and the obtained marine spatial patterns were not only well known but also were new to Earth scientists.

## Supporting information

S1 Dataset Monthly anomaly of sea-surface temperature (SSTA). (RAR)

S2 Dataset Monthly anomaly of sea level anomaly (SLAA). (RAR)

S3 Dataset Monthly anomaly of sea-surface precipitation (SSPA). (RAR)

S4 Dataset Monthly anomaly of sea-surface chlorophyll-a (CHLA). (RAR)

S1 Table Partial information on the mined frequent patterns in the Pacific Ocean. (DOCX)
