# Supplementary material for: Tree-based approach for exploring marine spatial patterns with raster datasets
Source: PLoS One. 2017 May 16;12(5):e0177438. doi: 10.1371/journal.pone.0177438 (PMC5433720; doi:10.1371/journal.pone.0177438)
Supplement: S1 Table — (DOCX) [file pone.0177438.s005.docx]

**S1 Table. Partial information on the mined frequent patterns in the Pacific Ocean**

| No | Space Index | Association Patterns | Support (%) | Confidence (%) | Lift |
| --- | --- | --- | --- | --- | --- |
| 743 | (1°N,173°E) | SSTA[-1]→ENSO[-1] | 19.44 | 85.37 | 2.44 |
| 744  745 | (1°N,173°E)  (1°N,173°E) | SSTA[+1]→ENSO[+1] | 14.44 | 83.87 | 2.70 |
|  |  | SLAA[+1]→CHLA[-1] | 12.22 | 81.48 | 3.33 |
| 746 | (1°N,173°E) | SSPA[-1]SSTA[-1] →ENSO[-1] | 13.33 | 96.00 | 2.74 |
| 747 | (1°N,173°E) | ENSO[+1]→CHLA[-1]SSTA[+1] | 11.11 | 90.91 | 2.92 |
| 748 | (1°N,173°E) | ENSO[+1]SLAA[+1]→CHLA[-1] | 10.56 | 95.00 | 3.87 |
| 749 | (1°N,173°E) | ENSO[+1]→CHLA[-1]SLAA[+1] | 10.56 | 86.36 | 2.78 |
| 750 | (1°N,173°E) | ENSO[-1]→CHLA[+1]SSPA[-1] | 12.22 | 91.67 | 2.62 |
| 809 | (1°N,174°E) | SSTA[-1]→ENSO[-1] | 20.56 | 86.05 | 2.46 |
| 810 | (1°N,174°E) | SSTA[+1]→ENSO[+1] | 13.89 | 83.33 | 2.68 |
| 811 | (1°N,174°E) | CHLA[+1]SSPA[-1]→ENSO[-1] | 11.17 | 90.91 | 2.58 |
| 812 | (1°N,174°E) | ENSO[+1]SLAA[+1] →CHLA[-1] | 12.29 | 95.65 | 4.18 |
| 813 | (1°N,174°E) | CHLA[-1]SLAA[+1] →ENSO[+1] | 12.29 | 91.67 | 2.98 |
| 814 | (1°N,174°E) | ENSO[-1]SSPA[-1] →SSTA[-1] | 15.56 | 96.55 | 2.76 |
| 871 | (1°N,175°E) | SSTA[+1]→ENSO[+1] | 11.67 | 84.00 | 2.70 |
| 872 | (1°N,175°E) | CHLA[-1]→ENSO[+1] | 21.23 | 86.36 | 2.76 |
| 873 | (1°N,175°E) | ENSO[-1]SSPA[-1] →SSTA[-1] | 15.00 | 93.10 | 2.66 |
| 874 | (1°N,175°E) | SSTA[-1]→ENSO[-1] | 20.00 | 83.72 | 2.39 |
| 875 | (1°N,175°E) | CHLA[-1]SLAA[+1]→ENSO[+1] | 12.29 | 91.67 | 2.93 |
| 876 | (1°N,175°E) | ENSO[-1] →CHLA[+1]SSPA[-1] | 12.85 | 82.14 | 2.33 |
| 877 | (1°N,175°E) | ENSO[+1]SLAA[+1]→CHLA[-1] | 12.29 | 95.65 | 3.89 |
| 754 | (0,173°E) | SSTA[-1]→ENSO[-1] | 20.00 | 85.71 | 2.45 |
| 755 | (0,173°E) | ENSO[-1] →SSPA[-1]SSTA[-1] | 11.67 | 91.30 | 2.61 |
| 815 | (0,174°E) | SSTA[-1]→ENSO[-1] | 20.00 | 85.71 | 2.61 |
| 816 | (0,174°E) | CHLA[-1]→ENSO[+1] | 20.67 | 80.43 | 2.57 |
| 817 | (0,174°E) | ENSO[-1] →SSPA[-1]SSTA[-1] | 13.33 | 88.89 | 2.54 |
| 818 | (0,174°E) | CHLA[-1]SLAA[+1]→ENSO[+1] | 11.17 | 100.00 | 3.20 |
| 819 | (0,174°E) | ENSO[+1]SLAA[+1]→CHLA[-1] | 10.61 | 86.36 | 3.36 |
| 820 | (0,174°E) | CHLA[+1]SSPA[-1]→ENSO[-1] | 10.61 | 95.00 | 3.04 |
| 878 | (0,175°E) | SSTA[-1]→ENSO[-1] | 20.56 | 82.22 | 2.35 |
| 879 | (0,175°E) | CHLA[-1]→ENSO[+1] | 22.22 | 83.33 | 2.68 |
| 880 | (0,175°E) | SSPA[+1]→ENSO[+1] | 14.44 | 81.25 | 2.61 |
| 881 | (0,175°E) | ENSO[-1] →SSPA[-1]SSTA[-1] | 15.00 | 84.38 | 2.41 |
| 882 | (0,175°E) | CHLA[-1]SSTA[+1]→ENSO[+1] | 11.11 | 95.24 | 3.06 |
| 883 | (0,175°E) | ENSO[+1]SSPA[+1]→CHLA[-1] | 12.22 | 84.62 | 2.68 |
| 884 | (0,175°E) | CHLA[-1]SSPA[+1]→ENSO[+1] | 12.22 | 100.00 | 3.21 |
| 885 | (0,175°E) | ENSO[+1]SLAA[+1]→CHLA[-1] | 11.11 | 83.33 | 3.13 |
| 886 | (0,175°E) | CHLA[-1]SLAA[+1]→ENSO[+1] | 11.11 | 95.24 | 3.06 |
| 887 | (0,175°E) | ENSO[+1]SSTA[+1]→CHLA[-1] | 11.11 | 83.33 | 3.13 |
| 759 | (1°S,173°E) | SSTA[-1]→ENSO[-1] | 19.44 | 83.33 | 2.38 |
| 760 | (1°S,173°E) | SSPA[-1]SSTA[-1]→ENSO[-1] | 10.56 | 82.61 | 2.36 |
| 823 | (1°S,174°E) | SSTA[-1]→ENSO[-1] | 19.44 | 81.40 | 2.33 |
| 824 | (1°S,174°E) | SSPA[-1]SSTA[-1]→ENSO[-1] | 11.11 | 86.96 | 2.48 |
| 890 | (1°S,175°E) | SSPA[-1]SSTA[-1]→ENSO[-1] | 11.11 | 90.91 | 2.60 |
| 891 | (1°S,175°E) | CHLA[-1]SSTA[+1]→ENSO[+1] | 10.61 | 95.00 | 3.04 |
| 892 | (1°S,175°E) | CHLA[-1]SSPA[+1]→ENSO[+1] | 12.85 | 92.00 | 2.94 |
| 893 | (1°S,175°E) | ENSO[+1]SSPA[+1]→CHLA[-1] | 12.85 | 88.46 | 3.30 |
